# Can single knockouts accurately single out gene functions?

**DOI:** 10.1186/1752-0509-2-50

**Published:** 2008-06-18

**Authors:** David Deutscher, Isaac Meilijson, Stefan Schuster, Eytan Ruppin

**Affiliations:** 1 Current address : Google Haifa, Haifa, Israel 31905; 2 School of Mathematical Sciences, Tel Aviv University, Tel-Aviv, 69978, Israel; 3 Faculty of Biology and Pharmaceutics, Section of Bioinformatics, Friedrich Schiller University Jena, Ernst-Abbe-Platz 2, D-07743 Jena, Germany; 4 School of Computer Sciences and School of Medicine, Tel Aviv University, Tel-Aviv, 69978, Israel

## Abstract

**Background:**

When analyzing complex biological systems, a major objective is localization of function – assessing how much each element contributes to the execution of specific tasks. To establish causal relationships, knockout and perturbation studies are commonly executed. The vast majority of studies perturb a single element at a time, yet one may hypothesize that in non-trivial biological systems single-perturbations will fail to reveal the functional organization of the system, owing to interactions and redundancies.

**Results:**

We address this fundamental gap between theory and practice by quantifying how misleading the picture arising from classical single-perturbation analysis is, compared with the full multiple-perturbations picture. To this end we use a combination of a novel approach for quantitative, rigorous multiple-knockouts analysis based on the Shapley value from game theory, with an established *in-silico *model of *Saccharomyces cerevisiae *metabolism. We find that single-perturbations analysis misses at least 33% of the genes that contribute significantly to the growth potential of this organism, though the essential genes it does find are responsible for most of the growth potential. But when assigning gene contributions for individual metabolic functions, the picture arising from single-perturbations is severely lacking and a multiple-perturbations approach turns out to be essential.

**Conclusion:**

The multiple-perturbations investigation yields a significantly richer and more biologically plausible functional annotation of the genes comprising the metabolic network of the yeast.

## Background

A central objective of the analysis of complex systems is localization of function, that is, determining which task is executed by each element or, more precisely, assessing how much each element contributes to the execution of specific tasks. For example, one can ask, for a given cell, which gene-products are responsible for energy metabolism, and which are involved in cell cycle and cell fate decisions. Many of the analysis techniques that are aimed at identifying the functional role of elements in biological systems, such as gene expression microarrays, are based on studying correlations in the data. However, establishing causal relationships in the data is not possible using purely correlational measures [1,2] without utilizing a randomized experimental framework [3]. For example, when observing a gene whose over-expression is coupled with a phenotypic phenomenon, one cannot ascertain whether the over expression is the direct cause of this phenomenon or an epiphenomenon of the activation of other genes.

These observations naturally lead to the notion of perturbations or knockouts: To understand what a specific element (gene, enzyme, neuron, brain area) does, lesion it and observe the functional consequences. A classical example is studying the effects of systematic deletions of genes on an organism's viability, with the resulting causal categorization of essential genes [4-6]. These classical knockout studies are however based on single-perturbations: In each experiment, a single element in the system is perturbed, and the resulting phenotype is used to identify its function. Yet, such single-perturbations will fail to reveal the functional organization of systems in which there is no one-to-one correspondence between elements and functions. For example, in a system with two redundant elements backing each other's function, the removal of any of the two will have no phenotypic effect, leading to the false conclusion that both elements are superfluous. Indeed, in a recent *in-silico *multiple-knockouts study of the robustness of the yeast's metabolic network [7], 74% of the genes were attributed some functional contribution, as opposed to only 13% found to be essential using single knockouts. There are a number of recent *in-silico *studies on the robustness against double and multiple knockouts (e.g., [8,9]).

Such considerations and findings suggest that understanding even moderately-complex biological systems requires the use of multiple concurrent perturbations. On the other hand, the vast majority of gene knockout studies employ only single knockouts. How detrimental is this gap? To what extent can we rely on the large body of observations that have been made using single knockout studies in biology?

The first obvious step toward addressing this question lies in double knockout perturbations. As the simplest conceivable genetic interaction is that of full overlap or redundancy (e.g., the result of gene duplication), one can set out to experimentally test the phenotype of all double concurrent perturbations, in search of gene pairs whose deletion is lethal (called *synthetic lethal*) or damaging. In fact, a large scale experiment looking for such gene pairs in *S. cerevisiae *[10] tested some 600,000 gene pairs and found ~4000 synthetic lethal pairs, at 0.65% frequency. These included ~1000 individual genes (almost all non-essential by themselves), encompassing about 16% of the genome. Two recent papers performed all double knockouts of yeast and the bacterium *Helicobacter pylori *metabolic genes using *in-silico *models [11,12].

These numbers offer a bipolar view on the importance of genetic interactions, and hence multiple-perturbations analysis, in functional genomics. On the one hand, one may convincingly argue that genes with complete or partial backups are in fact less important to the functioning of the organism, as each can be removed with small effect, and the probability of random mutations damaging both copies is very low. The rarity of synthetic lethal pairs is a further possible argument against the importance of multiple perturbations as a tool for localizing function. On the other hand, as only 20–30% of the yeast genes were found to be essential or partially contributing to growth (e.g., [5]), a multiple-knockouts analysis may expand considerably the effective size of the network of genes with significant functional contribution. In addition, more complex forms of redundancy and interactions are not necessarily manifested in synthetically lethal gene pairs. Finally, the functional annotations gained from single perturbations alone might be seriously lacking.

Travelling down this path of thought, the next obvious step is to experimentally test triple knockouts, and then quadruple and more. As these experiments are difficult and costly, the key question is whether these multiple-knockouts data will enhance our understanding of the system, and if so – to what extent. In a previous work [7], we have applied multiple concurrent knockouts to a widely accepted large scale *in-silico *model of the metabolism of *S. cerevisiae*, and enumerated synthetic lethal pairs and larger lethal groups termed *essential sets*. In this paper, we start by estimating the extent of multiple-lethality phenomena in *S. cerevisiae *using the same model, in a manner analogous to the experimental results cited. While useful in its own sake, we will see that this brute force approach leads to serious technical and conceptual scaling problems. Tackling these difficulties, we utilize a novel approach for quantitative rigorous multiple-knockouts analysis, the Multiple-perturbations Shapley value Analysis (MSA, See Methods), a methodology introduced at [13]. This method borrows fundamental concepts and analytical approaches from the field of Game Theory, which have already been used in many diverse fields [14-17].

The MSA utilizes perturbation (or knockout) experiments for assigning each element of a system a numerical contribution score (contribution value, CV) to a specific given task. Given such contributions we can address questions regarding the functional role of elements, the identification of submodules in the system, the quantification of localization/distribution of specific tasks, etc. The MSA is the first method providing a unique, axiomatically correct and scalable attribution of contributions to the system's elements, in the context of multiple-perturbations experiments. It was previously applied to brain networks, genetic networks and artificial neurocontrollers [18-20]. Using the MSA, we further analyze the yeast's metabolism to get more insight into the localization of metabolic function, and quantify how misleading the picture arising from classical single-perturbation analysis is in comparison.

## Results

### A Computational Study of Synthetic Lethality

Our first jab at the question of the necessity of multiple perturbations is an obvious one. We wish to estimate the extent of multiple-lethality phenomena in similar lines to the experiments of Tong et al. [10], that systematically crossed mutations in ~130 different query genes into a set of ~4700 viable gene yeast deletion mutants and identified synthetically lethal or sick interactions. To this end we employ the large scale *in-silico *constraint-based modelling approach of [21].

**Figure 1:**
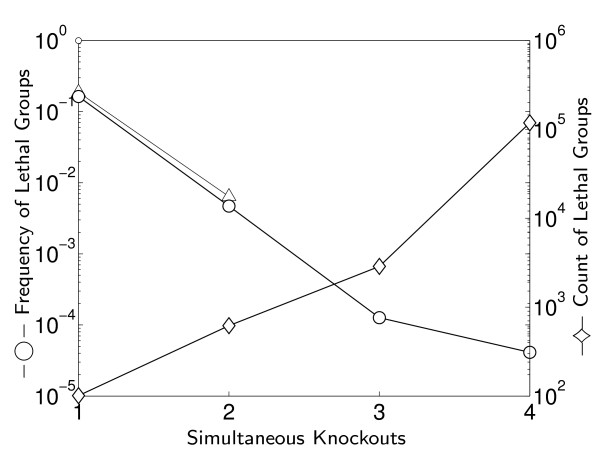
**How many synthetic lethals are there?**. For each perturbation size (knockout depth) we plot: -○- the frequency of lethal gene interactions, left axis; and -◇- the absolute number of lethal interactions, right axis. For sizes 1,2 we use the true numbers, while 3,4 are based on 2 · 10^6^ samples each (about 10% and 0.1% of the total interactions space, respectively). For all sizes we only count lethal gene groups for which no subgroup is in itself lethal. Compare with -△- for experimentally derived frequencies [5,10] for 1D and 2D interactions.

We have used the *in-silico *Flux Balance Analysis (FBA) model of [22] to predict the viability of *S. cerevisiae *under single knockouts of all genes included in the model, finding 101 essential genes comprising 16% of the 619 genes involved in the metabolic network modelled (see Methods). Limited only by computing power, we have proceeded to measure the viability of the yeast in the model under all double knockouts of non-essential genes, and under random samples of triple and quadruple knockouts. Figure 1 shows the observed frequency and count of lethal interactions per group size (essential genes, synthetic lethal pairs etc.).

Note the good agreement of the model statistics with the experimental data for the single and double knockouts: For knockout depths 1 and 2, the predicted lethal frequencies are 13% and 28%, respectively, lower than the empirical frequencies, a fairly small bias that might stem from the inherent optimism of the FBA predictions. Similarly, the statistics of three and four concurrent knockout experiments probably bound the expected true experimental frequencies from below, and probably not tightly so.

Both experimental and model data suggest that some important knowledge can be mined only from the multiple-perturbations experiments. Unfortunately, Figure 1 also points at two serious problems with this straightforward approach of measuring the amount of lethal interactions. First, it does not scale: On the one hand, as we use more concurrent perturbations, there is an *increasing *number of lethal interactions that we should aim to find. On the other hand, their frequency among all potential gene combinations is *decreasing*, because the number of such potential combinations grows even faster. Thus, even the switch to *in-silico *simulations, while accelerating the process over *in-vivo *experiments by many orders of magnitude, cannot provide a brute force complete answer to the multiple-perturbations problem. The second problem is a conceptual issue. Given the results of Figure 1, is it important to execute a multiple-perturbations analysis? Is the amount of interactions found small or large? How should one interpret the 200,000 four-dimensional interactions found in the metabolic network?

More specifically, as we use more concurrent perturbations two main problems potentially hinder the execution of the multiple-perturbations analysis: (a) without prior knowledge about the groups of elements involved in important interactions, the analysis requires the collection of an exponentially large (in the number of elements) set of costly multiple-knockouts experiments; and (b) it necessitates an accepted definition of importance for the analysis of the resulting large data set. In the following, we utilize the Multiple-perturbations Shapley value Analysis (MSA) method (see Methods) to overcome these issues.

### MSA Analysis of the Yeast Metabolic Network

Applying multiple concurrent perturbations to the metabolic network, we utilize MSA to assess the increase in information gained in the process. MSA, explained in more detail under Methods below, tackles the problem of quantifying the relative contributions of system elements (e.g., genes or enzymes) to a given task. The contribution is computed by measuring the effect of removing an element (e.g., gene deletion) not only in the fully functional system – as is done in classic single knockouts, but also after already removing one or more other elements. Thus, interactions between elements can be revealed. This basic idea was already employed, e.g., to identify synthetic lethal gene pairs [10] – but MSA introduced the computational and theoretical framework allowing the combination of many multiple knockout tests into a concise contribution score per element (for a given task). In out case, the elements perturbed are the genes coding for enzymes catalyzing the metabolic pathways. Perturbations are defined as complete knockouts of genes. The network's performance is the optimal solution found using FBA.

#### General Analysis: The Growth Task

Initially, we use the classic biomass production as the objective function in the FBA modelling. The biomass production target has proved successful in predicting experimental results in wild-type strains and mutants in several organisms [23-26]. It is also the objective function that best fits the experimental flux data among several functions tested in [27]. Although it is not necessarily the true single objective of the organism [28], maximization of growth is a good *in-silico *measure of the potential of growth of the organism.

**Figure 2:**
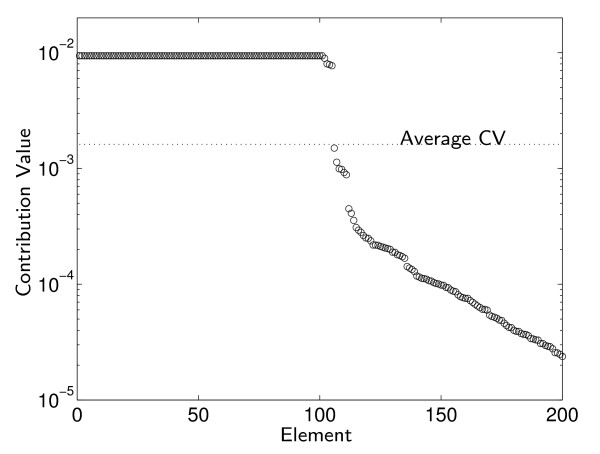
**Gene contribution values**. Gene contribution values for the general biomass production task of *S. cerevisiae *(ordered by decreasing contribution, first 200 genes shown). The high CV plateau marks essential genes, all equally important and cumulatively responsible for much of the growth.

Figure 2 presents the contribution values (CVs) of the 619 genes in the *S. cerevisiae *model, for the task of maximal growth. The distribution spans several orders of magnitude, which is to be expected: in accordance with the intuitive notion of importance discussed in the Introduction, the MSA attaches only small contribution values to redundant elements, as their contribution is marginal as long as the element buffering their function is intact. Importantly, however, it does attach non-zero contributions to them, in contrast to what might be expected from single knockout studies.

To compare the picture of function localization arising from a single perturbations analysis with that of the full MSA, we track 3 measures: (a) The number of genes involved in the task (requiring statistical significance, see Table 1); (b) The cumulative contribution of the essential (lethal) genes, i.e. the sum of their CVs; and (c) The mean relative error in the CVs computed using single-perturbations versus their true axiomatic value, as computed by an MSA of the multiple-perturbations data. Additionally, we record the number of essential genes for every task. Here we use the usual definition of essentiality, i.e., a gene is essential if its single-perturbation is lethal.

**Table 1:** Results of single vs. multiple perturbations analysis.

	As revealed by single knockouts	As revealed by multiple knockouts
(a) System size	131	215
(b) Cumulative CV of essential genes	96%	94.8%
(c) Mean relative error	45%	
(d) Number of essential genes	101	

(a) Number of genes implicated as involved in the task. In the multiple case, we only count genes with a statistically significant CV (corrected for multiple testing by FDR [37]: the expected proportion of falsely counted genes is controlled at 10%, or 22 in this case); (b) Total contribution of the essential genes; (c) Mean relative error of CVs; $MRE=\frac{1}{\left|I\right|}\cdot \sum_{i\in I}\left|\frac{\gamma_{i}^{\left(1\right)}-\gamma_{i}^{\left(n\right)}}{\gamma_{i}^{\left(n\right)}}\right|$ averaging only for the involved genes *I*; (d) Number of essential genes.

From the results presented in Table 1, one can derive two main conclusions regarding the localization of the general growth task: (a) the classic single-perturbation analysis misses some 33% of the genes found to have a statistically non-negligible contribution by the MSA. Since we used a conservative statistical estimation of the number of involved genes, this percentage might be even higher in the real organism. (b) Yet, the genes found by single knockout analysis contribute up to 96% of the cumulative sum of contributions to growth, while the many genes missed by the single-perturbation analysis end up having a very minor overall contribution in the MSA.

#### Production Of Biomass Constituents

We turn to a detailed localization of function, measuring the metabolic network's ability to individually produce the different biomass constituents. To this end we used an array of objective functions, each corresponding to the maximization of the production of a single biomass constituent, including the various amino acids, nucleotides, carbohydrates and lipids. We thus measure the contribution of genes to the potential production capability of each biomass constituent individually, in an isolated manner.

**Figure 3:**
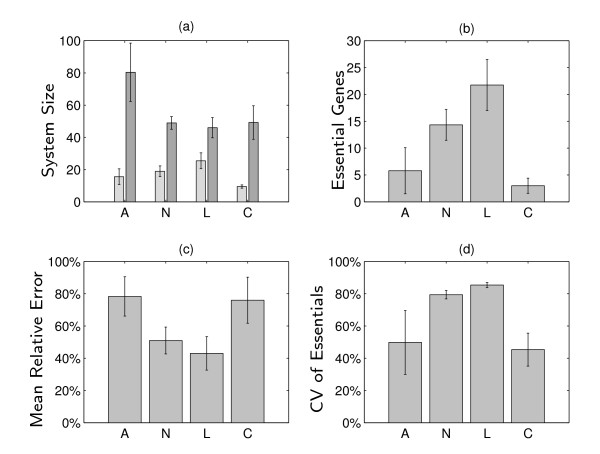
**Complexity of metabolic subsystems**. Considering 4 groups of tasks, for the production of the various biomass constituents: 20 **A**mino acids, 9 **N**ucleotides, 8 **L**ipids and sterols and 4 **C**arbohydrates. Mean/standard deviations across group members: **(a) **Count of genes implicated for involvement in the task (c.f. Table 1), left/right histograms mark the genes identified using single-perturbations/MSA respectively; **(b) **Number of essential genes; **(c) **Mean Relative Error (c.f. Table 1); **(d) **Total contribution of the essential genes.

Figure 3 applies the same measures as in Table 1 for the different tasks studied. It shows an important quality of the different subsystems in the metabolic networks: their respective complexity (which in this paper refers specifically to "localization complexity", i.e., the need for analyzing the system with multiple perturbations). It is clear that the amino-acid production systems are both larger and more complex (with functional overlap masking the discovery of involved genes) than the other groups, with lipid production being somewhat simpler than the other groups (Figures 3a and 3c). Concurrently, the amino acid and carbohydrate pathways have a significantly smaller number of essential genes (Figure 3b) compared with the other pathways. Single knockouts are only able to accurately recover the contributions of elements with little or no genetic interactions (like the essential genes), and indeed, when these elements are responsible for most of the task, single knockouts are quite accurate (Figures 3c and 3d). Most importantly, while the CVs of essential genes (revealed already by a single-perturbations analysis) cover about 95% of total CV mass in the general growth task (Table 1), it covers only a small percentage in some of the individual tasks, testifying to the necessity of multiple perturbations for correctly localizing the processing of individual tasks (Figure 3d).

**Figure 4:**
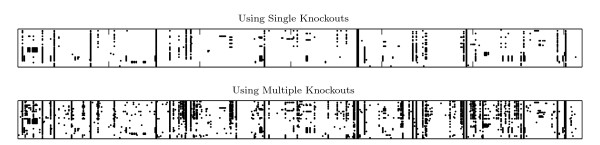
**Annotation matrices**. 41 × 619 element matrices, with dots indicating the gene (column) participates in the execution of the task (row). With multiple knockouts, only statistically significant genes are plotted (c.f. Table 1).

Hence, a multiple-knockouts analysis finds many more significant genes when examining individual tasks, than a single-knockout analysis. Figure 4 plots the genes (columns) participating in every task (rows) in both cases. The multiple-knockouts analysis provides a very large enrichment of the genes participating in each task and vice versa. For example, the single-knockout analysis functionally annotates only 148 genes by finding them important in one or more tasks (with 970 specific gene-per-task annotations (i.e., non-zero entries in the matrix)), while the multiple-knockouts analysis annotates 341 genes with a total of more than 2600 annotations. In other words, according to the multiple-knockouts analysis, each gene participates in many more tasks and one obtains a much richer, "soft" annotation of the functional roles of the gene products in the system. This conceptual picture conforms intuitively much better with the numerous annotations that genes may be ascribed to in standard annotation systems like GO, than the very sparse, "hard" annotation obtained with the single-lesion analysis.

**Figure 5:**
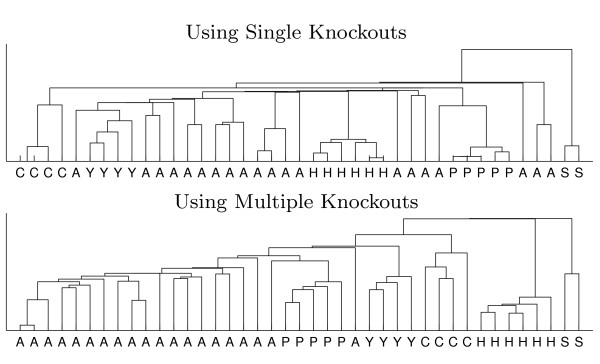
**Clustering of tasks by participating genes**. Hierarchical clustering of the 41 subtasks according to the binary vectors of participating genes from Figure 4. Clustering with a standard agglomerative, shortest single link algorithm, using a Jaccard distance metric with Matlab software. Tasks are manually classified according to product biochemical identity, to illuminate the clustering qualities: **A**mino acids, **P**urines, p**Y**rimidines, p**H**ospholipids, **S**terols and **C**arbohydrates.

To address the question whether the richer, multiple-knockouts annotation actually carries more valid biological information than the single-knockout analogue, we perform a hierarchical clustering of tasks, represented by their gene annotation vectors given in the data presented in Figure 4. The resulting cluster hierarchies in both the single and multiple knockouts cases are shown in Figure 5. Both clusterings successfully identify a few of the primary classes of the metabolic tasks (e.g., phospholipids and sterols). However, as evident, multiple-knockouts annotation produces a superior clustering, e.g., grouping correctly the amino acids and the pyrimidines to separate categories, while the single-knockout clustering fails to do so.

## Discussion and Conclusion

We present a large-scale study of function localization in a metabolic network model of the yeast. First, let us address our main question whether multiple knockouts are necessary to correctly localize function in simple organisms. We find that the essential elements revealed by the single-perturbations analysis of general growth span most of the total contribution values (CV mass). Yet, this analysis completely misses a large number (one third) of functionally relevant elements whose contribution, though small, is non-negligible and depends on the status of other elements. In the individual metabolic subtasks, the picture portrayed by single-perturbations analysis is significantly lacking, and in some of these subtasks the single perturbation analysis reveals only 20% of the contributing genes, which together account for only 40% of the total CV mass. The general growth task hence appears simpler than many of the subtasks producing biomass constituents. This is likely because it is a logical AND combination of the subtasks (all constituents are necessary for growth). This translates to an abundance of lethal elements in the general growth task, leaving relatively little mass (in the contribution sense) to the intricate interactions and nonlethal elements.

We began our investigation by mimicking *in-silico *the experimental paradigm recently introduced *in-vivo*, of performing a set of multiple knockouts (double, in the *in-vivo *case) and assessing the amount of lethal interactions that is uncovered. Remarkably, the model's results for the single and double knockouts concord fairly well with the *in-vivo *results. The triple and quadruple model experiments confirm the trends observed in the lower-dimensional interactions – that is, as the dimension (size) of the interaction increases, the frequency of lethal interactions decreases and their absolute number increases by about an order of magnitude. However, we find that this approach cannot be extended in practice, even by using *in-silico *models, because of two main reasons: First, it is computationally intractable, and second, and perhaps more important, it is not clear how the results obtained can actually be transcribed to measures of system size or complexity, or to yield a more useful functional gene annotation. We hence turn to a more rigorous, systematic approach. Using the MSA to quantitatively identify the significant genes and their contribution to the different metabolic tasks, we are able to characterize the functional profile of the genes in the system in both the single and multiple knockouts cases. Indeed, the multiple-knockouts analysis is shown to give a description which is both richer and conforms better with basic biological knowledge than the single knockout analysis.

In our multiple-knockouts study of the yeast metabolism, a batch of 10^5 ^perturbation experiments already gives a fairly accurate function localization picture for some tasks, in a system with 600 elements. Depending on the complexity of the analyzed system, knockout depths from *k *= 4 and up to *k *= 10 are needed. Given that current *in-vivo *experimental studies already perform tens of thousands of single perturbations [4] and hundreds of thousands of double-knockout experiments [10], it is not unreasonable to expect that an *in-vivo *multiple-perturbations analysis of large metabolic pathways, and perhaps even whole cellular systems, will become feasible in the foreseeable future.

One should note that the genes that require multiple knockouts to unravel their functional contributions are not of marginal importance and are of considerable biological significance, as already shown in previous studies: [7] have provided numerous examples of genes sets discovered by high-depth knockouts and discuss their backups from a biological perspective. [29] have shown that including the contributions of genes identified via double knockouts significantly extends the coverage and quality of their functional annotation in the yeast (vs GO and vs the annotation obtained considering only single knockouts). Specific examples of such genes are discussed in depth and the pathways that they compose are elucidated and further validated via auxotrophy experiments.

Summarizing our findings, this paper shows two fundamental results. First, in response to our basic research question, we show, for the yeast's metabolic network, that the conventionally used single knockout analysis provides only a very partial picture of function localization. Second, we show that the current experimental paradigm for probing the system's higher-order lethal interactions is both practically and conceptually limited. In turn, we demonstrate the essential value of a rigorous multiple-knockouts analysis method for accurately estimating the network's effective significant size. Moreover, the identification of the significant elements might even guide the experimental search for lethal interactions in the future, and make it more efficient. The current study of functional genomics in biological systems is just the beginning of an important endeavor, and much remains to be done. Furthermore, the multi-perturbation approach presented here is not necessarily limited to perturbations at the gene level. One potentially important extension is to the study of complex, combinatorial gene regulation programs, to elucidate the relative role of the different transcriptions factors and their binding sites on a given gene promoter. First steps in this direction have been recently taken by [30]. Overall, our results strongly indicate that multiple-knockouts experimental studies are likely to drastically change the way we understand and think about function localization in biological systems.

## Methods

### Constraint Based Models and Flux Balance Analysis

Genome sequencing and annotation have enabled the reconstruction of genome-scale metabolic networks. We use constraint-based models of *S. cerevisiae *[22]. These models enumerate the biochemical reactions involved and impose mass balance, thermodynamic and maximum flux constraints to define the set of flux vectors representing all possible steady states. Flux Balance Analysis (FBA) [31,32] is a constraint-based method which uses the objective function of maximum growth yield to find an optimal steady state in the set of feasible solutions. As a single optimal solution is rarely of interest, FBA is used to explore the optimal solution as a function of varying conditions. Several useful predictions have been obtained from such *in-silico *models [23,24], including predicting the consequences of gene deletions, optimal growth patterns, outcomes of adaptive evolution and more. The success rate of these predictions is typically in the order of 70–90% depending on the organism studied and the type of prediction being made. It is important to note that as optimality is sought, when the predictions are false these models tend to err in one direction more than the other: they are usually overly optimistic with regard to the organism's true capabilities, probably because maximum yield is never completely attained in Nature [25,28,33,34]. Briefly, the FBA method describes the stoichiometry of a system of *N *metabolic reactions involving *M *metabolites in an *M *× *N *matrix *S*. It then solves the following linear programming problem:

(1) $$\begin{array}{l} \max\;c^{T}v\\ \text{s}.\text{t}.\;Sv=0\\ \;l\leq v\leq u \end{array}$$

where *v *is the (unknown) vector of fluxes through the reactions. The constraints enforce a steady-state mass balance, and additional lower and upper bounds (*l *and *u*, respectively) are placed on some of the fluxes to enforce all irreversible reactions to have flux in the correct direction, limit environmentally available nutrients etc. At least one *v*_*i *_flux must be bounded from above in order to avoid diverging solutions, and because this is biologically meaningful, for example, due to limitation of substrate uptake. The constant vector *c *determines the optimization target. Here, we use different objective functions to enable the measurement of different functions or tasks, i.e., different aspects of metabolism. Specifically, we consider optimal production of biomass, and optimal production of particular biomass constituents. For the optimal biomass production in the yeast's case, FBA seeks the maximal production of a linear combination of biomass constituents, including 20 amino acids, 9 nucleotides, 8 lipids, phospholipids, fatty acids and sterols, and 4 carbohydrates, and sulfate (which is available from the environment). The weights for the linear combination were determined in [22]. We had simulated aerobic growth on minimal media based on Yeast Nitrogen Base (YNB) w/o amino acids, with glucose as carbon source.

### MSA: Multiple-Perturbations Shapley Value Analysis

Assume you can measure the system's performance at some task (e.g., the organism's growth rate), and that you can introduce multiple perturbations to the system before measuring performance. This is the essence of knockout studies (though we stress quantification while traditionally the results are often categorized). Obviously, for a system of *n *elements (genes) you can end up with 2^*n *^numbers. Even restriction to double perturbations solely yields ~*n*^2 ^results. This data set must be concisely summarized to be of any use. A basic summary should give each gene a contribution score, quantifying its importance to the successful performance of the task. This process can be repeated for different tasks, yielding a *soft annotation *vector for each gene, across the tasks, denoting to which tasks does it contribute in a significant manner. Another important consideration in looking for such a one-dimensional summary is scalability -these contribution values (CV) should be computable given a very partial data set, as the entire set of 2^*n *^experiments can hardly ever be accomplished in reality. The MSA [13] addresses the fundamental challenge of defining and calculating the contributions of network elements to a defined measurable task, given a data set of multiple-perturbations experiments and their corresponding performance scores.

To understand the MSA, examine a system of *N *= {1, ..., *n*} elements, performing a defined task. Suppose we measure the performance of the system at this task under all possible multiple perturbations, i.e., we have a performance score *v*(*S*), for every subset *S *⊆ *N *designating the unperturbed elements. Let the marginal contribution of element *i *to a group *S*, with *i *∉ *S*, be

(2) Δ_*i*_(*S*) = *v*(*S *∪ {*i*}) - *v*(*S*).

The contribution of element *i *∈ *N *as defined by the Shapley Value [35] (which is one of a broader family of semi-values differing in the way experiments are weighted; it is used for its axiomatic qualities, though our results remain qualitatively similar for other members of the semi-values family) is

(3) $$\gamma_{i}=\frac{1}{n!}\sum_{R\in R}\Delta_{i}(S_{i}(R))$$

where R is the set of all *n*! orderings of *N*, and *S*_*i*_(*R*) is the set of elements preceding *i *in the ordering *R*. The Shapley value is the unique fair division of the total performance gap *v*(*N*) - *v*(∅) among the different elements [35], i.e.

(4) $$\sum_{i=1}^{n}\gamma_{i}=v(N)-v(\emptyset ).$$

In this respect, the intuitive interpretation of the Shapley value is the relative fraction that each element plays in the total execution of the task. Note that *v*(*S*) can represent the performance after some transformations. For example, if one assumes a multiplicative effects model, it is reasonable to use a logarithm transform (as was done for example in [36]). While in traditional game theory the Shapley value is more a theoretical tool, the MSA introduces sampling methods to compute the CVs approximately with high accuracy and efficiency from a relatively small set of experiments.

Important to our work is the concept of *k *perturbations contribution value (*k*p-CV), which is a generalization of the CV concept, defined as

(5) $$\gamma_{i}^{\left(k\right)}=\frac{1}{\left|K\right|}\sum_{R\in K}\Delta_{i}(S_{i}(R))$$

where K={R∈R|R(i)>n−k} and *R*(*i*) is the position of element *i *in the permutation *R*, i.e. K includes exactly those orderings where *i *is in one of the last *k *positions. For *k *= *n *this reduces to the contribution value, while for *k *= 1 it is the result of the single-perturbation measurements. The *k*p-CV has a simple intuitive meaning; it measures the importance of elements when only *k*-limited knockout experiments (i.e., where no more than *k *elements are silenced concomitantly) are applied to the system. The *k*p-CV, computed with *k *= 1 enables one to assign contribution values to the elements and quantify their importance even using single-perturbations solely.

### Applying MSA

We utilize MSA to analyze the computational model of the yeast's metabolic network. The elements perturbed are the genes coding for enzymes catalyzing the metabolic pathways. Perturbations are defined as complete knockouts of genes. The network's performance is the optimal solution found using FBA. As directed by the MSA method, we sampled random orderings of the elements, then sequentially perturbed them in this order, and measured the performance after each consecutive perturbation. The MSA gives statistical error estimates that allow one to stop sampling when the accuracy of results is satisfactory [13]. These estimates usually converge faster for the dominant elements, with higher relative errors for the less important ones, which naturally lends to finding the former quicker. Depending on the specific experiment, we had used samples of 10^5 ^– 10^6 ^perturbation experiments to estimate the contributions of the genes, obtaining very small estimation errors. Unless explicitly marked, standard deviations of presented results are not shown, to reduce clutter.

## Authors' contributions

DD ran the simulations and carried out the data analysis. All authors contributed to the final manuscript.

## References

[B1] Pearl J (2000). Causality Models, Reasoning, and Inference.

[B2] Pe'er D, Regev A, Elidan G, Friedman N (2000). Inferring subnetworks from perturbed expression profiles. Bioinformatics.

[B3] Chen L, Emmert-Streib F, Storey J (2007). Harnessing naturally randomized transcription to infer regulatory relationships among genes. Genome Biol.

[B4] Carpenter AE, Sabatini DM (2004). Systematic Genome-Wide Screens Of Gene Function. Nat Rev Genet.

[B5] Giaever G, Chu A, Ni L, Connelly C, Riles L, Véronneau S, Dow S, Lucau-Danila A, Anderson K, André B, Arkin A, Astromoff A, El-Bakkoury M, Bangham R, Benito R, Brachat S, Campanaro S, Curtiss M, Davis K, Deutschbauer A, Entian K, Flaherty P, Foury F, Garfinkel D, Gerstein M, Gotte D, Güldener U, Hegemann J, Hempel S, Herman Z, Jaramillo D, Kelly D, Kelly S, Kötter P, LaBonte D, Lamb D, Lan N, Liang H, Liao H, Liu L, Luo C, Lussier M, Mao R, Menard P, Ooi S, Revuelta J, Roberts C, Rose M, Ross-Macdonald P, Scherens B, Schimmack G, Shafer B, Shoemaker D, Sookhai-Mahadeo S, Storms R, Strathern J, Valle G, Voet M, Volckaert G, Wang C, Ward T, Wilhelmy J, Winzeler E, Yang Y, Yen G, Youngman E, Yu K, Bussey H, Boeke J, Snyder M, Philippsen P, Davis R, Johnston M (2002). Functional profiling of the Saccharomyces cerevisiae genome. Nature.

[B6] Winzeler E, Shoemaker D, Astromoff A, Liang H, Anderson K, Andre B, Bangham R, Benito R, Boeke J, Bussey H, Chu A, Connelly C, Davis K, Dietrich F, Dow S, El Bakkoury M, Foury F, Friend S, Gentalen E, Giaever G, Hegemann J, Jones T, Laub M, Liao H, Liebundguth N, Lockhart D, Lucau-Danila A, Lussier M, M'Rabet N, Menard P, Mittmann M, Pai C, Rebischung C, Revuelta J, Riles L, Roberts C, Ross-MacDonald P, Scherens B, Snyder M, Sookhai-Mahadeo S, Storms R, Ve'ronneau S, Voet M, Volckaert G, Ward T, Wysocki R, Yen G, Yu K, Zimmermann K, Philippsen P, Johnston M, Davis R (1999). Functional characterization of the S. cerevisiae genome by gene deletion and parallel analysis. Science.

[B7] Deutscher D, Meilijson I, Kupiec M, Ruppin E (2006). Multiple knockout analysis of genetic robustness in the yeast metabolic network. Nat Genet.

[B8] Kuepfer L, Sauer U, Blank L (2005). Metabolic functions of duplicate genes in *Saccharomyces cerevisiae*. Genome Res.

[B9] Behre J, Wilhelm T, von Kamp A, Ruppin E, Schuster S (2008). Structural robustness of metabolic networks with respect to multiple knockouts. J Theor Biol.

[B10] Tong A, Lesage G, Bader G, Ding H, Xu H, Xin X, Young J, Berriz G, Brost R, Chang M, Chen Y, Cheng X, Chua G, Friesen H, Goldberg D, Haynes J, Humphries C, He G, Hussein S, Ke L, Krogan N, Li Z, Levinson J, Lu H, Ménard P, Munyana C, Parsons A, Ryan O, Tonikian R, Roberts T, Sdicu A, Shapiro J, Sheikh B, Suter B, Wong S, Zhang L, Zhu H, Burd C, Munro S, Sander C, Rine J, Greenblatt J, Peter M, Bretscher A, Bell G, Roth F, Brown G, Andrews B, Bussey H, Boone C (2004). Global Mapping of the Yeast Genetic Interaction Network. Science.

[B11] Segrè D, DeLuna A, Church G, Kishony R (2004). Modular epistasis in yeast metabolism. Nat Genet.

[B12] Thiele I, Vo T, Price N, Palsson BØ (2005). An expanded metabolic reconstruction of *Helicobacter pylori* (iIT341 GSM/GPR): an in silico genome-scale characterization of single and double deletion mutants. J Bacteriol.

[B13] Keinan A, Sandbank B, Hilgetag C, Meilijson I, Ruppin E (2004). Fair attribution of functional contribution in artificial and biological networks. Neural Comput.

[B14] Roth A (1979). Axiomatic models of bargaining.

[B15] Feigenbaum J, Papadimitriou C, Shenker S (2001). Sharing the cost of multicast transmisions. Journal of Computer and System Sciences.

[B16] Gefeler O, Land M, Eide G (1998). Averaging atributable fractions in the multifactorial situation: Asumptions and interpretation. J Clin Epidemiol.

[B17] Shubik M (1985). Game theory in the social sciences.

[B18] Keinan A, Sandback B, Kaufman A, Sachs N, Hilgetag C, Ruppin E (2004). Fair Localization of Function via Multi-lesion Analysis. Neuroinformatics.

[B19] Kaufman A, Keinan A, Meilijson I, Kupiec M, Ruppin E (2005). Quantitative analysis of genetic and neuronal multi-perturbation experiments. PLoS Comput Biol 2005 Nov;1(6):e64.

[B20] Keinan A, Sandbank B, Hilgetag C, Meilijson I, Ruppin E (2006). Axiomatic Scalable Neurocontroller Analysis Via the Shapley Value. Artif Life.

[B21] Price N, Papin J, Schilling C, Palsson BØ (2003). Genome-scale microbial in silico models: the constraints-based approach. Trends Biotechnol.

[B22] Förster J, Famili I, Fu P, Palsson BØ, Nielsen J (2003). Genome-Scale Reconstruction of the Saccharomyces cerevisiae Metabolic Network. Genome Res.

[B23] Famili I, Förster J, Nielsen J, Palsson BØ (2003). Saccharomyces cerevisiae Phenotypes can be Predicted using Constraint-based Analysis of a Genome-scale Reconstructed Metabolic Network. Proc Natl Acad Sci U S A.

[B24] Edwards J, Ibarra R, Palsson BØ (2001). *In silico* predictions of *Escherichi coli* metabolic capabilities are consistent with experimental data. Nat Biotechnol.

[B25] Förster J, Famili I, Palsson BØ, Nielsen J (2003). Large-scale evaluation of *in silico* gene deletions in *Saccharomyces cerevisiae*. OMICS.

[B26] Edwards J, Palsson BØ (2000). The *Escherichia coli* MG1655 *in silico* Metabolic Genotype: Its Definition, Characteristics, and Capabilities. Proc Natl Acad Sci U S A.

[B27] Burgard A, Maranas C (2003). Optimization-based framework for inferring and testing hypothesized metabolic objective functions. Biotechnol Bioeng.

[B28] Schuster S, Fell D, Lengauer T (2007). Modelling and simulating metabolic networks. Bioinformatics: From Genomes to Therapies.

[B29] Shlomi T, Herrgard M, Portnoy V, Naim E, Palsson BØ, Sharan R, Ruppin E (2007). Systematic condition-dependent annotation of metabolic genes. Genome Res.

[B30] Yosef N, Kaufman A, Ruppin E (2006). Inferring functional pathways from multi-perturbation data. Bioinformatics.

[B31] Varma A, Palsson BØ (1994). Metabolic Flux Balancing: Basic Concepts, Scientific and Practical Use. Bio/Technology.

[B32] Kauffman K, Prakash P, Edwards J (2003). Advances in flux balance analysis. Curr Opin Biotechnol.

[B33] Segrè D, Vitkup D, Church G (2002). Analysis of optimality in natural and perturbed metabolic networks. Proc Natl Acad Sci USA.

[B34] Shlomi T, Berkman O, Ruppin E (2005). Regulatory on/off minimization of metabolic flux changes after genetic perturbations. Proc Natl Acad Sci USA.

[B35] Shapley L, Kuhn H, Tucker A (1953). A Value for n-Person Games. Contributions to the Theory of Games.

[B36] Elena S, Lenski R (1997). Test of synergistic interactions among deleterious mutations in bacteria. Nature.

[B37] Benjamini Y, Hochberg Y (1995). Controlling The False Discovery Rate – A Practical And Powerful Approach To Multiple Testing. J Roy Stat Soc B Met.

